# Insect Floral Visitors of *Ptelea trifoliata* (Rutaceae) in Iowa, United States

**DOI:** 10.1093/aesa/saac012

**Published:** 2022-07-08

**Authors:** A J Talcott Stewart, M E O’Neal, W R Graves

**Affiliations:** Department of Horticulture, Iowa State University, 2206 Osborn Drive, 106 Horticulture Hall, Ames, IA 50011, USA; Department of Entomology, Iowa State University, 2310 Pammel Drive, Science Hall II, Ames, IA 50011, USA; Department of Horticulture, Iowa State University, 2206 Osborn Drive, 106 Horticulture Hall, Ames, IA 50011, USA

**Keywords:** dioecious, fruit-set, pollination, woody-plant

## Abstract

*Ptelea trifoliata* L., is a North American tree that supports insect communities through floral rewards. Our objectives were to determine the importance of insects as pollinators of *P. trifoliata*; describe the community of floral visiting insects of *P. trifoliata* in Iowa, where no such information was available; and to note insect preferences for male or female flowers. Over two years, inflorescences on 13 trees were covered with mesh bags before blooming and the amount of fruit produced was compared to uncovered inflorescences from the same trees. In one year, insects were collected from male and female trees with an insect vacuum every 3 h between 7 am and 7 pm from four sites in Iowa, USA between 30 May and 16 June 2020. In 2019 and 2020, almost no fruit set occurred from inflorescences covered with mesh bags while an average of 51.2 fruits formed on unbagged inflorescences (*P* < 0.0001), suggesting insects larger than the 600 μm pore diameters mesh were responsible for pollination of *P. trifoliata*. Insects from five orders, 49 families, and at least 109 species were collected. Most insects were Hymentoptera (48.3%) or Diptera (28.2%). Male flowers attracted 62.3% of all insects collected. Since most of the insects found visiting *P. trifoliata* were not bees, the floral rewards of the flowers may be a valuable resource for a wide variety of insects in the central United States.

Many plants provide ecosystem services such as purifying the air, absorbing storm water (Salmond et al. 2016), and supporting local communities of insects (Burghardt et al. 2009). Additionally, insect health is promoted by access to nectar and pollen from diverse and nutritious taxa (Huang 2012, Di Pasquale 2013). An example of a plant that could achieve these horticultural and ecological goals is *Ptelea trifoliata* L. or hop tree, a tree in the citrus family (Sapindales: Rutaceae). The native distribution of *P. trifoliata*, includes the Upper Midwest, but the species is infrequently promoted there for horticultural use (Bailey 1962, Yang and Applequist 2015, Dirr and Warren 2019). Its showy, fragrant flowers and glossy foliage are horticultural attributes, but the extent to which insects might benefit from increased cultivation of this species in the central United States had not been studied.

While not particularly economically valuable, *P. trifoliata* is ecologically important because it is utilized by several key insect species. The leaves of *P. trifoliata* are consumed by *Schistocerca emarginata* (=*lineata*) (Scudder) Vickery & D.K.M. Kevan (Orthoptera: Acrididae) and *Papilio cresphontes* Cramer (Lepidoptera: Papilionidae) (Scriber and Dowell 1991, Sword and Dopman 1999). In the northern parts of this butterfly’s distribution, *P. trifoliata* and *Zanthoxylum americanum* Mill. (Sapindales: Rutaceae) are the only known host plants and may be essential for the persistence of *P. cresphontes* in these areas. Larvae of *Agonopterix pteleae* Barnes & Busck and *A. costimacula* Clarke (Lepidoptera: Depressariidae), feed exclusively on *P. trifoliata* in Illinois (Harrison and Berenbaum 2005).

In Iowa, *P. trifoliata* blooms for about two weeks in late May and early June. Ambrose et al. (1985) has resolved the confusion about the reproductive biology of *P. trifoliata*, which had previously been called polygamodioecious (Bailey 1962, McLeod and Murphy 1977) and dioecious (Knuth 1908). Ambrose et al. (1985) observed that some female flowers had underdeveloped anthers without pollen and some male flowers had an underdeveloped pistil that did not produce seeds; however, the species was mostly functionally dioecious. This excepted 2–4% of male trees that had some complete flowers. The species has small greenish-white female flowers and creamy-white male flowers which form rounded inflorescences of 20–100 flowers (Figs. 1 and 2) (Ambrose et al. 1985). Flowers have a corolla comprised of five, slightly-pointed petals that give the flower an open, shallow-bowl shape about 1–1.5 cm across (Ambrose et al. 1985). Male flowers have four or five stamens with tricolporate pollen and female flowers have a pistil that begins to widen into a samara fruit before petals senesce. In the center of the fibrous, papery pericarp of the broadly winged, and indehiscent samaras are two cavities that each may contain one dark-colored seed (U.S. Department of Agriculture Forest Service 1948, Dirr 1998), though fruits containing only one seed are more common (Ambrose et al. 1985). Dioecious plants, like *Ptelea*, provide a unique situation because the composition or intensity of scents may differ between male and female flowers (Grison et al. 1999, Flamini et al. 2002). This sexual dimorphism has been shown to affect insects (Waelti et al. 2009). To what extant insect visitations to flowers of *P. trifoliata* varies by the sex of tree is not known.

**Fig. 1.**
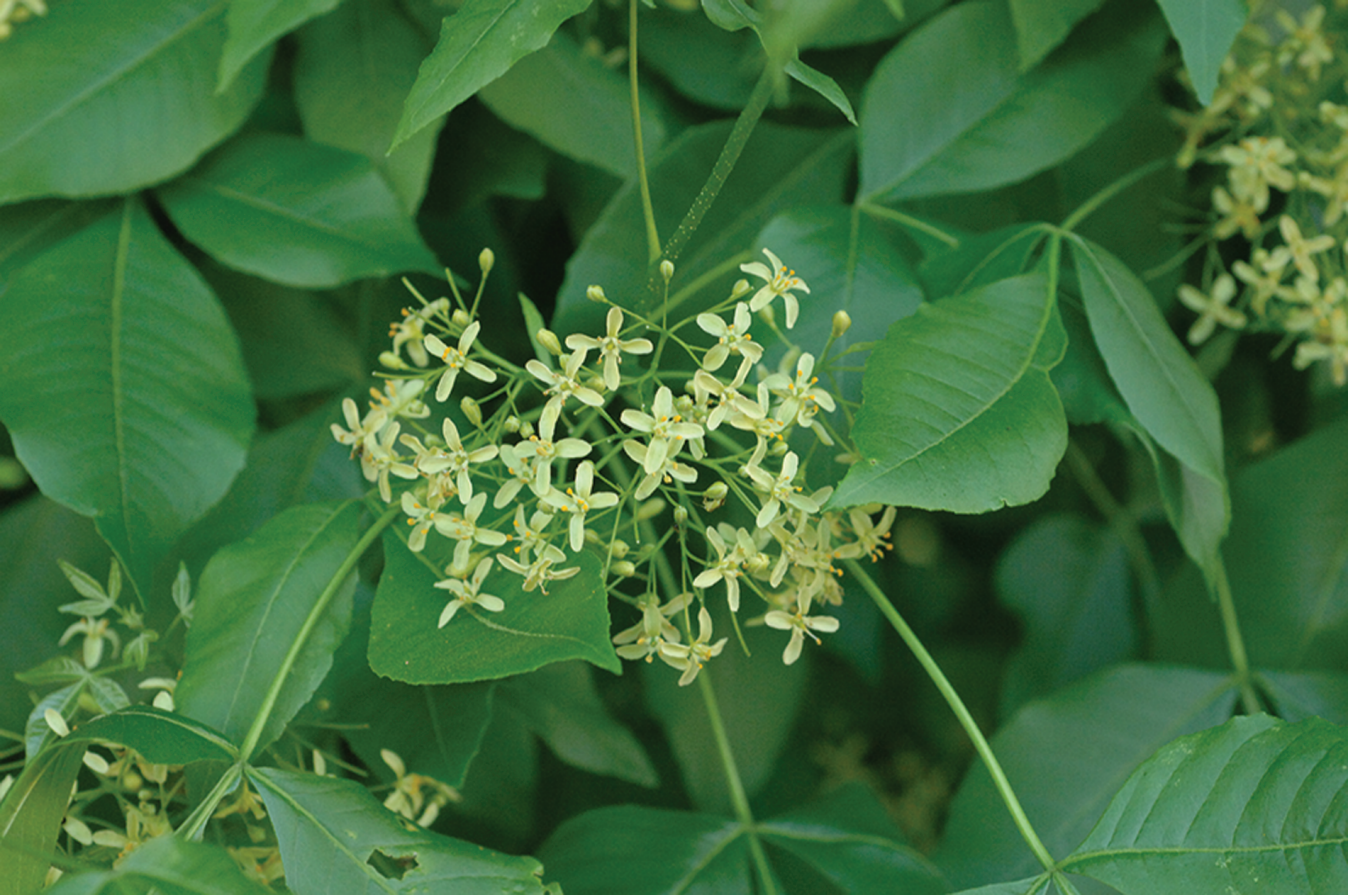
Picture of inflorescence of male flowers of *Ptelea trifoliata*. The shallow-bowl shape of the flower’s corolla is about 1–1.5 cm across. Male flowers have four or five stamens with tricolporate pollen. The difference in size between the pictured inflorescences in Figs. 1 and 2 is coincidental.

**Fig. 2.**
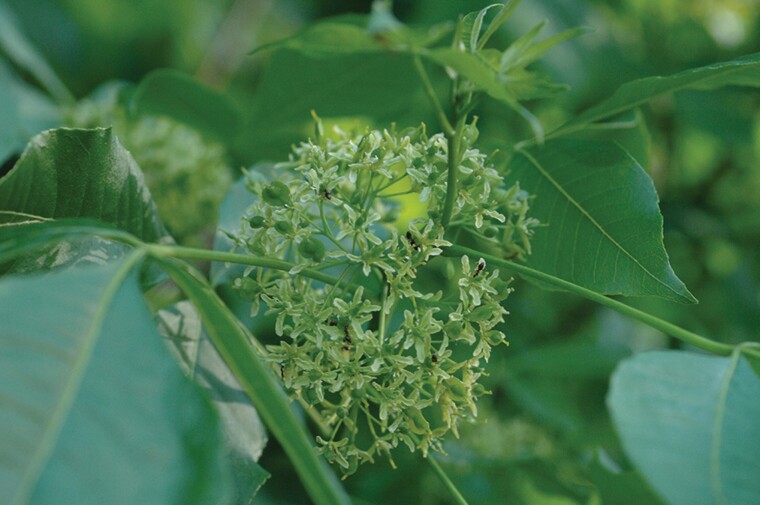
Picture of inflorescence of female flowers of *Ptelea trifoliata*. Green samaras, which will turn brown and papery as they mature, are developing as female flowers senesce. The difference in size between the pictured inflorescences in Figs. 1 and 2 is coincidental.

According to the pollination syndrome, small flowers with open, bowl-shaped, or flat forms that are clustered into inflorescences, such as *P. trifoliata*, attract insects with a broad host range (Proctor et al. 1996). In Ontario, Canada, *P. trifoliata* has been reported to attract 102 species from 40 insect families (Ambrose et al. 1985), but to what extent this community visits *P. trifoliata* in other regions of North America is not known. Ambrose et al. (1985) concluded that wind or small insects are responsible for pollinating a small percentage of female flowers within 2–15 m of a male tree, but that larger insects are primarily responsible for pollination.

Based on pollination syndromes and insect visitor information from sites in Ontario, Canada (Ambrose et al. 1985), we hypothesize that *P. trifoliata* is primarily pollinated by insects and that a broad number of insects visit the flowers. Our objectives were to estimate the importance of insect pollination for fruit set in *P. trifoliata*, to identify the taxa richness and abundance among floral visitors of *P. trifoliata* in Iowa near the geographical midpoint of its native distribution, and to compare the insect community found on male and female trees. We included both prairie and woodland study sites to represent the range of habitats that support *P. trifoliata* and its insect visitors.

## Materials and Methods

**Fig. 3.**
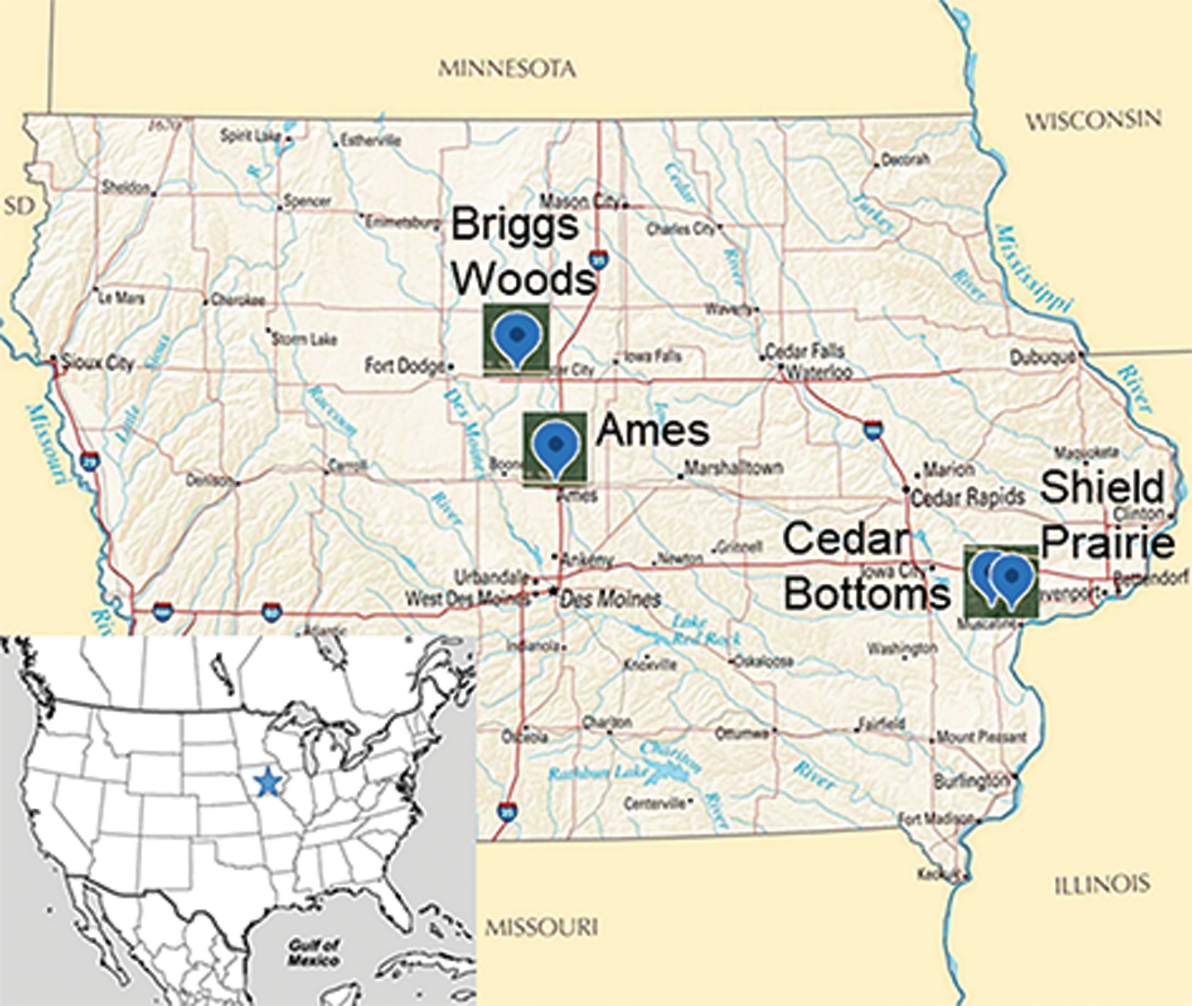
Location of the four sites in Iowa used for this study (Shield Prairie Wildlife Area in Muscatine County, Cedar Bottoms Wildlife Management Area in Muscatine County, Briggs Woods in Hamilton County, and Ames in Story County).

Four study sites were each visited several times between 30 May and 16 June 2020 for insect collection (Fig. 3). One was about 20 m along the edge of a woodland in Ames, Iowa (42.0141, −93.3920) with six female and 11 male trees. The second site was about 50 m^2^ of Briggs Woods in Hamilton County, Iowa (42.4361, −93.7948), with approximately 50 trees, at which we identified seven trees as females and 14 as males during flowering. The third was within Shield Prairie Wildlife Area near Muscatine, Iowa (41.4835, −91.1246), where 22 females and 20 males were dispersed over about 200 m^2^ of prairie. The final site was a 10-m^2^ wooded area in Cedar Bottoms Wildlife Management Area near Muscatine, Iowa (41.4653, −91.1905), with nine male trees and four females. We determined that managed colonies of honey bees (*Apis mellifera* L.) (Hymenoptera: Apidae) were within 1.5 km of the Ames and Shield Prairie sites, but were not near the Briggs and Cedar Bottoms sites, using the state of Iowa’s honey bee registry (FieldWatch, Inc. and Purdue Research Foundation 2021).

### Impact of Floral Insect Visitors on Fruit Set

On each tree, a nylon bag with 600 μm pore diameters mesh was placed over one female inflorescence with about the same number of buds as the rest of the inflorescences on the tree and secured with a plastic zip tie. One inflorescence was bagged from each of 10 trees of *P. trifoliata* at Shield Prairie on 28 May 2019 and three trees from Ames on 30 May 2020. The bag was intended to prevent insects larger than the mesh pores from pollinating the flowers, but still allow for wind pollination. The presence of each samara in the fall was considered evidence of successful pollination of one flower in the inflorescence. For each of the 13 trees, the number of samaras formed from each bagged inflorescence was compared to an unbagged inflorescence with about the same number of samaras as the rest of the inflorescences on the tree in the fall. Fruit number between bagged and unbagged inflorescences was compared with a *t*-test (JMP Pro 15 Software; version 15, SAS Institute Inc., Cary, NC).

### Insect Community

Insects that landed on flowers of *P. trifoliata* were collected with an insect vacuum (BioQuip Products, Inc., Rancho Dominguez, CA) five times during the day, every 3 h between 7 am and 7 pm. At each collection time, 20 min were devoted to collecting from female trees and 20 min from male trees. We counted those insects that were too numerous to collect. This included *Chaulignathus* sp. Hentz (Coleoptera: Cantharidae), *Macrodactylus* subspinosus Fabricius (Coleoptera: Scarabaeidae), and *Heliomata cycladata* Grote and Robinson (Lepidoptera: Geometridae) at Shield Prairie and ants crawling on flowers at the Ames site. Six females and 11 males were blooming at the Ames site on the collection dates (30 May, 1 June, and 7 June). Two to five females and two to four males were blooming at Shield Prairie on the collection dates (3 June, 11 June, and 15 June). We collected at Cedar Bottoms on the same days as Shield Prairie when three to seven males and zero to three females were blooming. No female trees were blooming on the last collection date at Cedar Bottoms. For these three sites, the three collection dates corresponded with early, peak, and late blooming. Seven females and 14 males were at peak bloom at Briggs Woods on the collection date 6 June; however, rain prevented us from collecting there further before the flowers senesced. Insects were kept in resealable plastic bags and frozen until pinned and identified. Insects were identified to the lowest taxonomic level possible with help from several keys (Skevington et al. 2019, Ascher and Pickering 2021, VanDyk 2021). Once an insect was identified, we noted the presence of pollen on its body. Voucher samples were deposited into the Iowa State University Insect Collection.

### Data Analysis

Fruit number between bagged and unbagged inflorescences was compared with a *t*-test (JMP Pro 15 Software). We report the taxa richness as the total number of the lowest taxonomic level present at a site. Shannon’s index and equitability were also calculated (Shannon 1948, Pielou 1966). We constructed sample-size based rarefaction and extrapolation curves for the insect communities visiting male and female trees with R (v. 4.0.3, R Development Core Team, Vienna, Austria) to determine if our sampling effort was sufficient to estimate the entire community. We used the vegan package (Community Ecology Package V2.4–6; Oksanen et al. 2018), the SpadeR package (Species-Richness Prediction and Diversity Estimation with R V1.1.1, Chao et al. 2016), and the INext package (Interpolation and Extrapolation for Species Diversity V2.0.12 2016, Hsieh et al. 2016) to construct and visualize the curves. A nonmetric, multidimensional scaling plot (NMDS) was constructed to visually compare the floral-visiting insect communities on male and female *P. trifoliata* using the ‘metaMDS’ function (Oksanen et al. 2018). We chose the Bray-Curtis metric in NMDS scaling because of its consideration of abundance. The stress value was 0.14 (not markedly above the 0.1 threshold), which confirmed that the resulting NMDS output plotted in a 2D plot sufficiently maintained the dissimilarities observed in the original data (Buja et al. 2008). A permutated multivariate analysis of variance (perMANOVA) tested the degree of similarity between floral insect communities on male and female trees at different sites. An analysis of variance (ANOVA) compared the mean number of insects on male and female trees.

## Results

An average of 0.15 ± 0.10 (standard error, SE) fruits occurred on inflorescences within mesh bags, while an average of 51.2 ± 6.74 (SE) fruits formed on uncovered inflorescences (*F* = 57.23; df = 1, 24; *P* < 0.0001). Both of the fruits that formed within the mesh bags contained one seed (with a potential maximum of two seeds).

Across all four locations, 2013 insects were identified, representing at least 109 species (Tables 1 and 2). Only 43% of the specimens collected were identified to species so the species richness may be higher than the reported taxa richness. Insect floral visitors included five orders with 48.2% hymenopterans, 28.2% dipterans, 14.4% lepidopterans, 7.7% coleopterans, and 1.5% hemipterans.

**Table 1.** Abundance and diversity indices of flower-visiting insects to *Ptelea trifoliata* organized by order and sex of the plant at four Iowa sites^*a*^

Ordersby sex of the tree	Shield Prairie	Cedar Bottoms	Briggs Woods	Ames	Total	Total (%)
**Coleoptera**	**145**	**3**	**2**	**4**	**154**	
Female	44	0	1	2	47	30.5
Male	101	3	1	2	107	69.5
**Diptera**	**233**	**90**	**162**	**83**	**568**	
Female	104	15	49	29	197	34.7
Male	129	75	113	54	371	65.4
**Hemiptera**	**1**	**5**	**2**	**23**	**31**	
Female	1	1	1	7	10	32.3
Male	0	4	1	16	21	67.7
**Hymenoptera**	**222**	**44**	**32**	**673**	**971**	
Female	58	5	9	366	438	45.1
Male	164	39	23	307	533	54.9
**Lepidoptera**	**283**	**2**	**0**	**4**	**289**	
Female	64	0	0	2	66	22.8
Male	219	2	0	2	223	77.2
**Grand total**	**884**	**144**	**198**	**787**	**2013**	
Female	271	21	60	406	758	37.7
Male	613	123	138	381	1255	62.3
Species richness	71	35	30	43		
Shannon’s index	1.83	3.04	2.36	1.84		
Equitability	0.43	0.84	0.69	0.48		

^*a*^Shield Prairie Wildlife Area in Muscatine County, Cedar Bottoms Wildlife Management Area in Muscatine County, Briggs Woods in Hamilton County, and Ames in Story County.

**Table 2.** Insects visiting flowers of *Ptelea trifoliata* collected or counted from four sites in Iowa sites^*a*^ and surveyed for the presence of pollen.

Taxa	Shield Prairie	Cedar Bottoms	Briggs Woods	Ames	Pollen present
COLEOPTERA	**145**	**3**	**2**	**4**	
**Cantharidae**	**48**	**1**	**0**	**0**	
Cantharinae	3	0	0	0	
*Chaulignathus*sp. Hentz	45	0	0	0	
unidentified	0	1	0	0	
**Cerambycidae**	**0**	**0**	**0**	**4**	
**Cleridae**	**1**	**0**	**0**	**0**	
*Enoclerus* sp. Gahan	1	0	0	0	
**Coccinellidae**	**1**	**1**	**0**	**0**	
*Harmonia* sp. Étienne Mulsant	1	0	0	0	
unidentified	0	1	0	0	
**Elateridae**	**1**	**0**	**1**	**0**	
**Lampyridae**	**0**	**1**	**0**	**0**	
**Scarabaeidae**	**94**	**0**	**1**	**0**	
*Dichelonyx* sp. Harris	0	0	1	0	
*Macrodactylus subspinosus* Fabricius	94	0	0	0	
DIPTERA	**233**	**90**	**162**	**83**	
**Anthomyiidae**	**79**	**26**	**87**	**19**	
Anthomyiidae sp. 1	51	20	65	13	
Anthomyiidae sp. 2	28	6	22	6	
**Bibionidae**	**2**	**1**	**0**	**0**	
*Bibio* sp. Geoffroy	1	1	0	0	
*Dilophus* sp. Meigen	1	0	0	0	
**Calliphoridae**	**2**	**4**	**0**	**9**	
**Conopidae**	**4**	**0**	**0**	**0**	
*Physocephala* sp. Schiner	4	0	0	0	
**Culicidae**	**4**	**2**	**1**	**0**	
**Dolichopodidae**	**1**	**0**	**0**	**0**	
**Hybotidae**	**2**	**0**	**0**	**0**	
*Platypalpus* sp. Macquart	2	0	0	0	
**Muscidae**	**3**	**12**	**1**	**0**	
**Platystomatidae**	**64**	**0**	**0**	**0**	
*Rivellia* sp. Robineau-Desvoidy	64	0	0	0	
**Polleniidae**	**6**	**10**	**0**	**0**	
*Pollenia* sp. Robineau-Desvoidy	6	10	0	0	
**Sarcophagidae**	**2**	**0**	**3**	**0**	
Miltograminae	0	0	1	0	
Sarcophaginae	0	0	1	0	
unidentified	2	0	1	0	
**Scathophagidae**	**2**	**0**	**0**	**0**	
*Scathophaga stercoraria*L.	2	0	0	0	
**Sepsidae**	**14**	**4**	**5**	**1**	
*Sepsis punctum* Fabricius	14	4	5	1	
**Stratiomyidae**	**3**	**7**	**0**	**0**	
**Syrphidae**	**41**	**23**	**65**	**48**	
Pipizinae	1	0	0	0	
Syrphinae	0	1	0	0	
*Allograpta obliqua* Say	4	2	31	2	*
*Chrysotoxum* sp. Meigen	0	0	2	0	
*Epistrophe* sp. Walker	0	0	1	0	*
*Eupeodes* sp. Matsumura	0	0	1	1	
*Platycheirus* sp. Lepeletier and Serville	1	0	1	0	*
*Sphaerophoria contigua* Macquart	3	2	0	1	
*Syrphus* sp. Fabricius	2	7	8	3	*
*Toxomerus geminatus* Say	0	2	19	13	*
*Toxomerus marginatus* Say	30	9	2	27	
*Volucella evecta* Walker	0	0	0	1	
**Tachinidae**	**2**	**0**	**0**	**0**	
*Gymnocheta* sp. Robineau-Desvoidy	1	0	0	0	
unidentified	1	0	0	0	
**Tipulidae**	**2**	**1**	**0**	**1**	
**Ulidiidae**	**0**	**0**	**0**	**5**	
*Delphinia picta* Fabricius	0	0	0	5	
HEMIPTERA	**1**	**5**	**2**	**23**	
**Lygaeidae**	**0**	**1**	**0**	**0**	
*Lygaeus turcicus* Fabricius	0	1	0	0	
**Miridae**	**1**	**4**	**2**	**20**	
*Ceratocapsus juglandis* Knight	0	0	1	17	
*Opistheurista clandestina* Van Duzee	0	2	1	0	
*Taedia* sp. Distant	1	0	0	0	
unidentified	0	2	0	3	
**Pentomidae**	**0**	**0**	**0**	**1**	
**Reduvidae**	**0**	**0**	**0**	**2**	
HYMENOPTERA	**222**	**44**	**32**	**673**	
**Andrenidae**	**113**	**11**	**17**	**86**	
*Andrena anograe* Cockerell	0	0	0	1	*
*Andrena barbilabris* Kirby	22	0	2	0	*
*Andrena brevipalpis* Cockerell	61	9	10	71	*
*Andrena commoda* Smith	2	0	0	0	*
*Andrena crataegi* Robertson	2	0	0	0	*
*Andrena cressonii cressonii* Robertson	0	0	0	1	*
*Andrena forbesii Robertson*	1	0	0	0	*
*Andrena haynesi* Viereck and Cockerell	1	1	0	1	*
*Andrena hirticincta* Provancher	0	0	0	2	
*Andrena ilicis* Mitchell	14	1	0	6	*
*Andrena illini* Bouseman and LaBerge	0	0	0	2	*
*Andrena illinoiensis* Robertson	1	0	0	0	*
*Andrena imitatrix* Cresson	1	0	0	0	*
*Andrena milwaukeensis* Graenicher	1	0	0	0	*
*Andrena miranda* Smith	4	0	4	1	*
*Andrena sigmundi* Cockerell	1	0	0	1	*
*Andrena surda* Cockerell	0	0	1	0	*
*Andrena wheeleri* Graenicher	1	0	0	0	*
*Andrena wilkella* Kirby	1	0	0	0	*
**Apidae**	**3**	**2**	**0**	**17**	
*Ceratina* sp. Latreille	0	1	0	8	
*Nomada pygmaea* Cresson	0	0	0	1	
*Nomada* sp. Latreille	3	1	0	8	
**Argidae**	**3**	**0**	**0**	**0**	
*Arge humeralis* Beauvois	3	0	0	0	
**Braconidae**	**1**	**2**	**1**	**0**	
**Colletidae**	**4**	**0**	**0**	**0**	
*Hylaeus* sp. Fabricius	4	0	0	0	
**Crabronidae**	**2**	**0**	**0**	**1**	
Bembicinae	1	0	0	0	
Philanthinae	1	0	0	0	
*Anacrabro ocellatus* Packard	0	0	0	1	
**Formicidae**	**1**	**2**	**0**	**461**	
*Camponotus* sp. Mayr	1	0	0	0	
unidentified	0	2	0	461	
**Halictidae**	**20**	**7**	**11**	**76**	
*Augochlora pura* Say	0	0	1	0	*
*Augochloropsis sumptuosa* Smith	0	0	1	0	*
Augochlorini	0	0	0	2	*
*Halictus* sp. Latreille	0	1	0	0	*
*Lasioglossum (Dialictus)* sp. Robertson	3	0	8	70	*
*Lasioglossum (Dialictus)* *nigroviride* Graenicher	0	0	0	4	
*Lasioglossum (Dialictus) nymphale* Smith	11	5	0	0	*
*Lasioglossum (Evylaeus)* sp. Robertson	2	0	1	0	*
*Sphecodes* sp. Latreille	4	1	0	0	*
**Ichneumonidae**	**4**	**0**	**0**	**0**	
Pimplini	4	0	0	0	
**Pompilidae**	**1**	**0**	**0**	**0**	
Pepsinae	1	0	0	0	
**Rhopalosomatidae**	**1**	**0**	**0**	**0**	
**Sphecidae**	**3**	**0**	**0**	**3**	
*Ammophila* sp. Host	1	0	0	1	
*Isodontia apicalis* Smith	1	0	0	0	
*Isodontia* sp. Patton	0	0	0	2	
unidentified	1	0	0	0	
**Tenthredinidae**	**1**	**0**	**0**	**0**	
**Vespidae**	**65**	**20**	**3**	**30**	
*Ancistrocerus unifasciatus* Saussure	0	0	0	4	
*Eumenes fraternus* Say	2	0	0	10	
*Polistes* sp. Latreille	2	0	0	0	
*Vespula flavopilosa* Jacobson	0	0	0	2	
Eumeninae	61	20	3	14	
LEPIDOPTERA	**283**	**2**	**0**	**4**	
**Crambidae**	**0**	**1**	**0**	**0**	
*Desmia* sp. Westwood	0	1	0	0	
**Geometridae**	**279**	**0**	**0**	**0**	
*Heliomata cycladata* Grote and Robinson	279	0	0	0	
**Lycaenidae**	**1**	**0**	**0**	**2**	
*Celastrina* sp. Wright and Pavulaan	1	0	0	2	
**Noctuidae**	**3**	**1**	**0**	**2**	
*Alypia* sp. Hübner	1	0	0	0	
unidentified	2	1	0	2	

^*a*^Shield Prairie Wildlife Area in Muscatine County, Cedar Bottoms Wildlife Management Area in Muscatine County, Briggs Woods in Hamilton County, and Ames in Story County.

Most of the hymenopterans that visited the flowers of *P. trifoliata* were from the Andrenidae, Formicidae, Halictidae, and Vespidae (Table 2). Of those identified to species, all were common North American natives. *Andrena brevipalpis* Cockerell (Hymenoptera: Andrenidae), the subgenus *Dialictus* Robertson from the genus *Lasioglossum* Curtis (Hymenoptera: Halictidae), and the wasp subfamily Eumeninae (Hymenoptera: Vespidae) were abundant and present at all sites (Table 2). Diptera abundant at all sites include two unidentified species of Anthomyiidae, along with *Sepsis punctum* Fabricius (Diptera: Sepsidae), *Allograpta obliqua* Say (Diptera: Syrphidae), and *Toxomerus marginatus* Say (Diptera: Syrphidae) (Table 2).

**Fig. 4.**
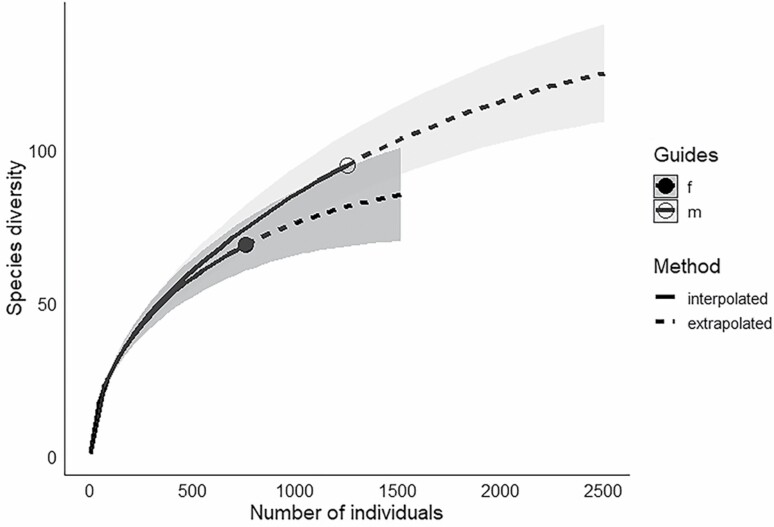
Sample-size-based rarefaction (solid lines) and extrapolation (dotted lines) curves with 95% confidence intervals (shaded) for insect visitors of female (f) and male (m) trees by species diversity (taxa richness).

**Fig. 5.**
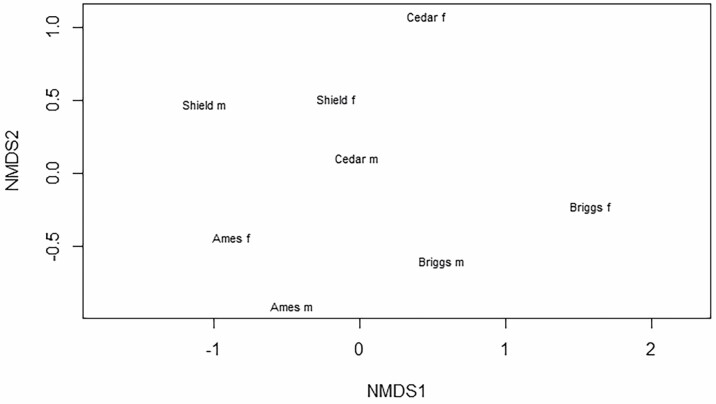
Nonmetric, multidimensional scaling plot of the floral-visiting insect communities on male (m) and female (f) trees of *Ptelea trifoliata* at Shield Prairie Wildlife Area (Shield), Cedar Bottoms Wildlife Management Area (Cedar), Briggs Woods (Briggs), and Ames.

Male flowers attracted 1,255 insect visitors, while females had 758 visitors (Table 1). The abundance of floral visitors to male and female trees was similar based on the ANOVA (*F* = 1.28; df = 1; *P* = 0.28). Rarefaction and extrapolation curves for visitors of male and female trees indicate that greater sampling effort would have increased the amount of additional taxa collected (Fig. 4). The NMDS plot, confirmed by the perMANOVA, indicated that floral-visiting insect communities of male and female trees of *P. trifoliata* were similar (*F* = 1.07; df = 1, 3; *P* = 0.45) and sites were different (*F* = 4.15; df = 3, 3; *P* = 0.004; Fig. 5). The Shield Prairie and Ames sites had the greatest taxa richness, 73 and 45 taxa, respectively (Table 1). Cedar Bottoms and Briggs Woods had the highest Shannon’s index and equitability, which measure evenness of the taxa diversity in a community (Table 1). Equitability rates the Shannon’s index, with 1 equaling the most even taxa diversity possible for a site. Ants in Ames and three taxa at Shield Prairie—*Chaulignathus* sp., *Macrodactylus* sp., and *Heliomata cycladata*—particularly reduced the evenness of these flower visitor communities.

Thirty-one of the taxa identified had pollen on their bodies. With the exception of *Andrena hirticincta* Provancher (Hymenoptera: Andrenidae) and *Lasioglossum (Dialictus) nigroviride* Graenicher (Hymenoptera: Halictidae), all the smallest taxonomic groups identified from Andrenidae and Halictidae had pollen on their bodies; a few syrphids also had pollen on their bodies (Table 2).

## Discussion

Based on the comparison of bagged versus unbagged flowers, *P. trifoliata* appears to be pollinated almost entirely by insects. The flowers of *P. trifoliata* attracted a wide variety of insects from five orders, 49 families, and 109 taxa (Tables 1 and 2). With the notable exceptions of *Chaulignathus* sp., *Macrodactylus subspinosus*, and *Heliomata cycladata*, most insect visitors were flies, bees, and wasps (Tables 1 and 2). Many insects identified are common species native to North America. Based on the type of insects with pollen observed on them, solitary, native bees from Andrenidae and Halictidae, as well as flower flies (Syrphidae) may be the primary pollinators of *P. trifoliata*; however, further research confirming pollen identity is necessary to determine the pollinators of *P. trifoliata* (Table 2).

Similarly to Ambrose et al. (1985), almost no fruit set occurred within mesh bags in our study, indicating that insects too large to fit through the 600 μm pore diameter mesh in the bags are the main pollinators of *P. trifoliata*. The two fruits that did form within the mesh bags each contained one seed, which rules out the possibility of parthenocarpy, or production of seedless fruit without fertilization. *Ptelea trifoliata* is dioecious, but Ambrose et al. (1985) observed 2–4% of male plants had some flowers that developed fruit. This could explain how two flowers were fertilized within the mesh bags, beyond the possibility of wind pollination.

In Ontario, Canada, Coleoptera, Diptera, Hemiptera, and Hymenoptera have been reported visiting the flowers of *P. trifoliata*, but not Lepidoptera (Ambrose et al. 1985). We identified several of the same families as visitors, such as Syphidae, Calliphoridae, Anthomyiidae, Tachinidae, Andrenidae, Halictidae, Apidae, Formicidae, and Argidae; but Ambrose et al. (1985) also observed 38 honey bees (*Apis mellifera* L.), while we found none in spite of the fact that two sites were within 1.5 km of honey bee hives (FieldWatch, Inc. and Purdue Research Foundation 2021). At Shield Prairie we observed an abundance of three taxa not seen at our other sites or by Ambrose et al. (1985). *Chauliognathus* sp. or soldier beetles are diverse nectar and potentially pollen feeders (Williams 2006). Considered minor pests, *Macrodactylus subspinosus* feed on a variety of flowers and foliage (VanDyk 2021). *Heliomata cycladata* are nectar feeders and their larval hosts are locusts (*Robinia* L.) (Fabales: Fabaceae) (Wagner et al. 2001).

While our results indicate high diversity among floral visitors, rarefaction, and extrapolation curves indicate that even more diversity could have been observed with increased sampling effort (Fig. 4). Male flowers attracted 62.3% of insect visitor individuals, suggesting a preference for male flowers, though not significant statistically. Every order except Hymenoptera showed a stronger preference for male flowers than the overall percentage, though insects from every order visited both male and female flowers (Table 1). Insects’ preference for male flowers could be attributed to the additional pollen reward offered by male flowers or a difference in the fragrance profile between male and female flowers. Overlapping confidence intervals generated by the rarefaction curves indicate that the taxa richness on male and female plants was similar; however, it is possible that with further collection effort, the communities could prove to be more distinct based on the extrapolation (Fig. 4). NMDS plot and perMANOVA results also confirm that the floral visiting insect communities are similar between male and female trees (Fig. 5). Ambrose et al. (1985) spent equal time collecting insects from male and female trees and did not report an insect preference for either sex in *P. trifoliata* in Ontario. Shield Prairie and Cedar Bottoms are more similar to each other and Briggs Woods and Ames are more similar to each other, which may be related to the nearness of these two sets of sites geographically (Figs. 3 and 5). While Shield Prairie and Ames sites had the highest taxa richness, an abundance of a few taxa reduced the Shannon’s index and equability values below the corresponding values for Cedar Bottoms and Briggs Woods (Tables 1 and 2).

The large variety of insect visitors observed corresponds with a prediction based on pollination syndromes that certain floral traits present in *P. trifoliata* are associated with pollinators with a broad host range (Proctor et al. 1996). The shallow-bowl shape of small flowers allows for easy harvesting of pollen and nectar. High exposure to sunlight and air currents promotes evaporation around the inflorescences, which results in a highly viscous nectar solution that attracts short-tongued bees, flies, wasps, and beetles, which can easily reach the nectar source and have no difficulty drinking the highly concentrated nectar that open flowers produce (Proctor et al. 1996). These floral traits are consistent with those observed for *P. trifoliata.*

In contrast with this syndrome’s predictions, the flowers of *P. trifoliata* at Shield Prairie also attracted hundreds of the butterfly *Heliomata cycladata*, which would be expected to have trouble drinking viscous nectar (Proctor et al. 1996). While the accuracy of pollination-syndrome predictions is somewhat controversial, it is interesting to note that the pollinators of monoecious and dioecious plants, like *P. trifoliata*, have been more likely to be correctly predicted than those for plants with perfect flowers (Waser et al. 1996, Rosas-Guerrero et al. 2014).

Waser et al. (1996) observed that flowers that attract pollinators considered generalists are more common than flowers that attract specialists. Attracting specialist pollinators may seem superior to attracting generalists because the likelihood of successful pollination increases if insects are visiting fewer species; however, Waser et al. (1996) pointed out that many plants cannot ‘afford to’ depend on a single or small number of insect species that might shift temporally or spatially from year to year. This is especially true if a plant has a short reproductive period, which is true of *P. trifoliata.* Attracting a multitude of insects may also give generalist plants an advantage in adjusting to climatic change, as plant and insect species may not always migrate at the same rates or in the same directions (Bartomeus et al. 2011, Sgolastra et al. 2016).

In conclusion, *P. trifoliata* is pollinated almost entirely by insects, though a trace percentage may be pollinated by wind or very small insects. Both male and female flowers of *P. trifoliata* were visited by many insect taxa from five orders at four sites in Iowa. This research expands our understanding of the ecological role of *P. trifoliata.* The promotion of *P. trifoliata* could support a wide range of insects. While previous research has revealed the importance of *P. trifoliata* as an essential or nearly essential host plant for several lepidopterans such as *P. cresphontes*, *A. pteleae*, and *A. costimacula*; and as a food source for certain floral visitors, this study enhances our knowledge of floral visitors that are supported by *P. trifoliata* in Iowa near the center of its native range.
